# Parents With Mental Illness: Parental Coping Behavior and Its Association With Children's Mental Health

**DOI:** 10.3389/fpsyt.2021.737861

**Published:** 2021-10-18

**Authors:** Marlit Sell, Alina Radicke, Bonnie Adema, Anne Daubmann, Reinhold Kilian, Maja Stiawa, Mareike Busmann, Sibylle M. Winter, Martin Lambert, Karl Wegscheider, Angela Plass-Christl, Silke Wiegand-Grefe

**Affiliations:** ^1^Department of Child and Adolescent Psychiatry, Psychotherapy and Psychosomatics, University Medical Center Hamburg-Eppendorf, Hamburg, Germany; ^2^Department of Medical Biometry and Epidemiology, University Medical Center Hamburg-Eppendorf, Hamburg, Germany; ^3^Department of Psychiatry II, Ulm University at Bezirkskrankenhaus Guenzburg, Günzburg, Germany; ^4^Department of Child and Adolescent Psychiatry, Psychosomatics and Psychotherapy, Charité Universitätsmedizin Berlin, Corporate Member of Freie Universität Berlin, Humboldt-Universität zu Berlin, and Berlin Institute of Health (BIH), Campus Virchow-Klinikum, Berlin, Germany; ^5^Department of Psychiatry and Psychotherapy, University Medical Center Hamburg-Eppendorf, Hamburg, Germany; ^6^Department of Child and Adolescent Psychiatry, Evangelical Hospital Hamburg Alsterdorf, Hamburg, Germany

**Keywords:** coping, parental mental illness, child mental health, risk factors, protective factors, children of parents with a mental illness (COPMI)

## Abstract

The relevance of coping behavior for the individual's own mental health has been widely investigated. However, research on the association between coping of parents with a mental illness and their children's mental health is scarce. In the current study, we address the role of several parental coping strategies and their relation to child psychological symptoms. As part of the German randomized controlled multicenter study CHIMPS (children of mentally ill parents), parents with mental illness completed questionnaires on illness-related coping and child mental health symptoms. Children's diagnoses of a mental disorder were assessed with diagnostic interviews. The sample comprised *n* = 195 parents with mental illness and *n* = 290 children and adolescents aged 4–18 years. We conducted mixed models to investigate the associations of parental coping strategies with internalizing and externalizing symptoms as well as the diagnosis of a mental disorder in children controlling for sociodemographic factors and parental symptom severity. Parental coping characterized by religiosity and quest for meaning was significantly associated with fewer mental health symptoms and lower odds of a mental disorder in children, whereas a depressed processing style was related to increased internalizing problems in the children. Coping behavior in parents with mental illness is a relevant factor for the mental health of their children and should be considered in preventive interventions.

## Introduction

Approximately 17–28% of families worldwide involve a parent with mental health symptoms (1–3). Research has consistently demonstrated that children of parents with a mental illness (COPMI) have themselves an increased risk for mental health symptoms (4, 5). COPMI have a significant higher risk to develop any mental health symptoms compared to children of healthy parents (6–8). The elevated risk has been established for various mental disorders, including depression (9), anxiety disorders (10), schizophrenia (11), as well as personality disorders (12) and has been shown to persist into adulthood (13). Familial aggregation has also been reported for related clinical outcomes like suicidal behavior (14, 15). Furthermore, parental mental illness can be classified under adverse childhood experiences (16) which have frequently been linked to reduced physical and mental health (17).

Risk and protective factors of mental health symptoms in COPMI are linked to parents, children, and the social environment (18, 19). On the parent-level, mental health symptoms can be related to reduced quality and quantity of child-parent interactions as well as psychological mental health of the children (5, 6, 18, 20, 21). Parenting skills and the children's risk for mental health symptoms are associated with characteristics of parental mental illness, including symptom burden, comorbidity (10), severity (22), and duration of illness (23). On the familial and environmental level, the families of parents with mental illness face a number of practical and emotional challenges, that may include financial problems, worries about the patient, missed career opportunities, and family quarrels (24–26). The family burden associated with parental mental illness can be a major source of distress and diminished quality of life in children and adolescents (26, 27).

Whereas, some stressors that are associated with mental health symptoms are unavoidable, parental coping with stressful situations can be influenced e.g., by effective coping skills training (28, 29) and psychotherapeutic interventions for adults (30–32). Moreover, it is important to understand which coping behavior can be considered adaptive or maladaptive, because parental coping has significant implications not only for the parents but also for their children's mental health (33, 34). It can also help to develop effective psychological and supportive interventions for parents that target illness perceptions and promote adaptive coping strategies to ultimately improve the family's mental health and quality of life (18). Cognitive and behavioral coping strategies generally aim at reducing or managing situational demands that exceed one's resources (35). Coping efforts can target the stressor directly (problem-oriented coping), attempt to regulate emotions (emotion-focused coping), to seek social support, or to avoid threatening situations by engaging mentally and physically from the situation (36, 37). Coping with mental health symptoms and their broader implications is challenging and often a lifelong process (38). Parents with mental illness have to deal with the dual demands of mental illness and parenting. Coping challenges can be related to diagnosis and treatment, stigmatization, interpersonal difficulties, reduced social support, the strain of parenthood and custody issues (39).

Adaptive coping behaviors comprise making a plan of action to deal with mental illness, share experiences with others in similar situations, and emphasize one's strengths and positive attributes (40). Flexible and effective coping requires the patient to accept the mental diagnosis and to develop a positive attitude toward treatment and recovery (41, 42). Adaptive coping has been associated with less symptom severity over time, less symptom burden and comorbid symptoms as well as a shorter duration of mental illness (38). It further potentially mitigates the negative outcomes of family burden and stigmatization by the society that can cause psychological distress and lower quality of life in children (26). Maladaptive coping strategies include denial, self-blame, behavioral disengagement, venting, and substance use (40). Such strategies are linked to increased negative emotions and psychopathology and lower quality of life in patients (43) and also to compromised children's mental health. For example, parents' denial of mental illness constitutes a risk factor for adverse psychosocial outcomes in children (33, 44). A recent systematic review concluded that parental mental illness is often described by children as a family secret. Especially younger children are influenced by parents' policy of not talking to friends or other connections outside the family. Some are also not allowed to talk with other family members about the parental mental illness (45). Psychoeducation has become a popular component of interventions for children and their parents aiming to reduce guilt and shame related to parental mental illness and to improve the children's mental health (46, 47).

A number of contextual factors such as differences in mental diagnosis, symptoms, personality, cognitive function, social environment, and sociodemographic aspects (e.g., cultural and gender differences, family influences) affect how parents deal with their mental health symptoms (38). For example, some patients with schizophrenia increase their level of physical activity when coping with distress, whereas patients with depression usually experience a lack of energy and rather cope by modifying thought contents (38, 48). Furthermore, research has shown that the appraisal of a stressor is important for the selection of the coping strategy. Lazarus and Folkman (37) proposed that when people believe they can handle or change a situation, they are more likely to choose problem-oriented coping strategies. Coping efforts focusing on regulating emotions are more often used when the situation seems unchangeable for the individual. Cognitive biases that affect threat appraisal and coping efforts have been identified in various mental disorders. For example, people with anxiety disorders overestimate threats and underestimate coping resources (49). In addition, due to negative emotionality, parents with mental illness may generally have an impaired ability to recognize a wider range of possible coping strategies and are more likely to use maladaptive coping (50–52).

Previous research has mainly investigated associations between coping behavior and its impact on the individual's own mental health. Only a few studies have investigated the association between coping behavior of parents with mental illness and their children's mental health (53, 54). Those predecessor studies by Wiegand-Grefe et al. (53) and Wiegand-Grefe et al. (54) identified significant correlations between some parental coping strategies (active, problem-oriented coping, religiosity and quest for meaning, as well as a depressed processing style) and child mental health, whereby only active-problem oriented coping was associated with fewer psychological difficulties in children. However, those studies used bivariate analyses without considering possible confounding by other variables. The present study aimed at examining the association of several parental coping strategies with child mental health by controlling for parental psychological symptom burden and the family's sociodemographic characteristics. Based on the previous research, we expected that active, problem-oriented coping in parents will be associated with less psychopathology in children, whereas religiosity and quest for meaning, as well as a depressed processing style will be related to increased psychopathology.

## Materials and Methods

### Study Design

Baseline data of the German study ‘CHIMPS' (children of mentally ill parents) gathered between 2014 and 2017 were analyzed. The randomized controlled multicenter study “Implementation and evaluation of a family-based intervention program for children and adolescents of mentally ill parents” addressed families with parental mental illness. It aimed at evaluating the effectiveness of a family intervention in improving the family members' mental health and health-related quality of life. Details of the study are reported elsewhere (55). Parents with mental illness and their families were recruited in several German and Swiss psychiatric departments. Inclusion criteria included that at least one parent had a mental disorder according to ICD-10 diagnosed by a clinician. For the current analyses, children were included between the ages of 4 and 18 years. Further, consent to participate in the study and proper proficiency of the German language of parents and children were required. Exclusion criteria included severe psychiatric symptoms of parents requiring inpatient treatment. The Ethics Committee of the regional Medical Association (Hamburg) approved the study under the number PV4744. Written informed consent was obtained from all adult participants. Written assents of children (<18 years) and their parents' permission were provided.

### Participants

The overall CHIMPS project sample included 216 families with 216 parents with mental illness and 338 children. Families were excluded when children did not meet the questionnaires age criteria (*n* = 31 in 29 families) or had more than 30% questionnaire-related missing values (*n* = 17 in 12 families). The final sample consisted of 195 parents with mental illness and 290 children aged 4–18 years.

### Measures

#### Mental Health Symptoms of Children

The widely used Child Behavior Checklist 4–18 (56) measures maladaptive emotional and behavioral symptoms in children aged 4–18 years on a three-point response scale ranging from 0 = “not at all” to 2 = “often.” The parent proxy-version has 118 items and generates the two broad band scores externalizing and internalizing symptoms. Raw and T-scores were calculated according to the manual (57, 58). T-scores below 60 represent the normal range, T-scores between 60 and 63 the borderline clinical range and T-scores above 63 the clinical range. The questionnaire demonstrated good psychometric properties in numerous clinical and non-clinical samples (57, 58). In our sample, excellent internal consistency was established by the internalizing (Cronbach's α = 0.90) and externalizing subscale (Cronbach's α = 0.92).

Children's diagnoses of a mental health disorder were assessed with semi-structured interviews using the German version of the Kiddie Schedule for Affective Disorders and Schizophrenia for School-Age Children-Present and Lifetime Version [K-SADS-PL; (59, 60)]. Interviews were conducted by trained clinicians with the parent version of the instrument. Diagnoses are provided according to the DSM-IV-TR and ICD-10. For the present study, only current episodes of children's psychopathology were assessed. The K-SADS-PL is a widely used instrument with good psychometric properties (61, 62).

#### Mental Health Symptoms of Parents

The Brief Symptom Inventory [BSI; (63, 64)] measures the perceived burden of mental health symptoms. It has 53-items that are answered on a five-point response scale ranging from 0 = “not at all” to 4 = “extremely.” It generates a Global Severity Index (GSI) that assesses the subjective burden of current or past symptoms, as well as the number and intensity of symptoms. The BSI has good psychometric properties (63). In this study, the GSI demonstrated an excellent internal consistency (Cronbach's α = 0.96).

#### Parental Coping With Mental Illness

The Freiburg Questionnaire of Coping with Illness [FQCI; (65)] has 23 items and enables respondents to self-report their predominant coping behavior on a four-point response scale ranging from 1 = “not at all” to 5 = “very much.” The five subscales cover depressed processing style, active problem-oriented coping, distraction and self-growth, religiosity and quest for meaning, as well as trivialization and wishful thinking. The authors report an internal consistency of Cronbach's α = 0.68–0.77 depending on the subscale (65). In this study, the internal consistency ranged from Cronbach's α = 0.49 (religiosity and quest for meaning) to 0.72 (trivialization and wishful thinking).

### Data Analysis

To account for family as a cluster, we conducted all analyses with mixed models (66). We first compared COPMIs' internalizing and externalizing problems with children from the reference population (58). We then examined the association between parental coping and internalizing and externalizing problems in children as well as children's diagnosis of a mental disorder, controlling for the children's age and gender, the parents' gender and their psychological symptom burden. Grand-mean centering was used for all continuous regressors. Questionnaire-related missing values were imputed with the expectation-maximation algorithm (67). Statistical significance was set at *p* < 0.05. The linear mixed-models procedure in IBM SPSS 26 (IBM, Armonk, NY, USA) was used to analyze the associations with children's internalizing and externalizing problems. Associations with children's diagnosis were analyzed with a binary logistic mixed model using the “glmer” function of the “lme4” package in R version 4.1.0.

## Results

### Descriptive Statistics

Tables 1, 2 display the sample characteristics. The parental sample consisted of 195 parents (*n* = 146 mothers and *n* = 49 fathers with mental illness) with a mean age of 40.34 years (*SD* = 6.96). About half of the sample was married. On average, parents had two children with a mean age of *M* = 9.97 years (*SD* = 4.03) with whom most shared the same household. Most parents had at least 10 years of school education. The most prevalent primary mental ICD-10 diagnoses were mood (affective) disorders (F30–F39), followed by disorders of adult personality and behavior (F60–F69), neurotic, stress-related and somatoform disorders (F40–F48). Less prevalent were schizophrenia, schizotypal and delusional disorders (F20–F29), mental and behavioral disorders due to psychoactive substance use (F10–F19) and behavioral and emotional disorders with onset usually occurring in childhood and adolescence (F90–F98). Almost half of the sample had a comorbid mental diagnosis. Parents with mental illness had a mean BSI GSI of *M* = 1.34 (*SD* = 0.69). Most of them practiced active-problem oriented coping, followed by depressed processing style. Parents rated their children's internalizing problems with *M* = 12.47 (*SD* = 9.27) and children's externalizing problems with *M* = 12.50 (*SD* = 9.77) on the CBCL 4–18. Of the children, *n* = 117 (43.5%) met criteria for any mental disorder as indicated by the K-SADS-PL. Most diagnoses were neurotic, stress-related and somatoform disorders with the most prevalent specific diagnoses being adjustment disorders (F43.2, *n* = 35) and specific phobias (F40.2, *n* = 17).

**Table 1:** Characteristics of parents with a mental illness.

**Characteristics**	***n* (%)**	***M* (*SD*)**
**Sociodemographic data**
Age (in years)^1^		40.34 (6.96)
Female^1^	146 (74.9)	
Number of children^1^		1.95 (0.93)
Married^1^	109 (55.9)	
School education^1^		
11–13 years education	59 (31.6)	
10 years education	86 (46.0)	
9 years education	38 (20.3)	
No secondary education	4 (2.1)	
**Mental health**		
Psychiatric diagnosis (ICD-10)^2^, ^a^		
F10–F19	3 (1.5)	
F20–F29	8 (4.1)	
F30–F39	112 (57.4)	
F40–F48	25 (12.8)	
F60–F69	46 (23.6)	
F90–F98	1 (0.5)	
Comorbidity (ICD-10)^2^	79 (40.5)	
Psychological symptom burden (BSI)^1^		1.34 (0.69)
Parental coping (FQCI)^1^		
Depressed processing style		3.17 (0.75)
Active problem-oriented coping		3.19 (0.77)
Distraction and self-growth		2.82 (0.67)
Religiosity and quest for meaning		2.59 (0.67)
Trivialization and wishful thinking		2.61 (1.01)

*n = 195. Questionnaire-related scores were based on raw data*.

1 *Based on parent self-reports*.

2 *Based on proxy-ratings by clinicians*.

a *International Classification of Diseases (ICD-10) codes: mental and behavioral disorders due to psychoactive substance use (F10–F19), schizophrenia, schizotypal, and delusional disorders (F20–F29), mood (affective) disorders (F30–F39), neurotic, stress-related and somatoform disorders (F40–F48), disorders of adult personality and behavior (F60–F69), behavioral and emotional disorders with onset usually occurring in childhood and adolescence (F90–F98); BSI, Brief Symptom Inventory; FQCI, Freiburg Questionnaire of Coping with Illness*.

**Table 2:** Characteristics of children.

**Characteristics**	***n* (%)**	***M* (*SD*)**
**Sociodemographic data**
Age (in years)^1^		9.97 (4.03)
Female^1^	152 (52.4)	
Shared household with parent with MI^1^	257 (89.5)	
(Step) siblings^1^	221 (76.2)	
**Mental health**
Emotional and behavioral symptoms (CBCL 4-18)^1^		
Internalizing problems		12.47 (9.27)
Externalizing problems		12.50 (9.77)
Psychiatric diagnoses (K-SADS-PL, ICD 10)^2^, ^a^
F30–F39	18 (6.2)	
F40–F48	66 (22.8)	
F90–F98	64 (22.1)	
Comorbidity (K-SADS-PL, ICD 10) ^2^	43 (14.8)	

*n = 290. Questionnaire-related scores were based on raw data*.

1 *Based on parent proxy-reports*.

2 *Based on proxy-ratings by clinicians. MI, mental illness. CBCL 4-18, Child Behavior Checklist 4–18. K-SADS-PL = Kiddie Schedule for Affective Disorders and Schizophrenia for School-Age Children-Present and Lifetime Version*.

a *International Classification of Diseases (ICD-10) codes: mood (affective) disorders (F30–F39), neurotic, stress-related and somatoform disorders (F40–F48), behavioral and emotional disorders with onset usually occurring in childhood and adolescence (F90–F98)*.

Parents' proxy-reported significantly more internalizing and externalizing problems than the reference population (*M* = 50, *SD* = 10) with an adjusted mean *T*-value of 63.08 (95% CI = 61.62–64.54, *p* < 0.001) for internalizing problems and of 58.99 (95% CI = 57.63–60.36, *p* < 0.001) for externalizing problems.

### Associations of Parental Coping With Mental Illness and Their Children's Mental Health

The associations of parental coping and mental health in COPMI aged 4–18 years are displayed in Table 3 (internalizing symptoms), Table 4 (externalizing symptoms), and Table 5 (diagnosis of a mental illness). Religiosity and quest for meaning as well as depressed processing style were significantly associated with internalizing symptoms. Higher levels of religiosity and quest for meaning were associated with a decrease in internalizing symptoms, whereas a more depressed processing style was related to an increase in internalizing symptoms. The control variables age of children and psychological symptom burden in parents were also significantly associated with internalizing symptoms. Parents' proxy-reported more internalizing symptoms when children were older and when parents perceived a high psychological symptom burden.

**Table 3:** Association of parental coping with mental illness and internalizing problems in children and adolescents.

**Effects**	**Coefficients**	**95% CI**
**Fixed effects**
Intercept	12.92^***^	[11.45, 14.40]
Parental coping (FQCI)		
Depressed processing style	2.16^*^	[0.50, 3.82]
Active problem-oriented coping	0.80	[−0.81, 2.40]
Distraction and self-growth	1.36	[−0.50, 3.23]
Religiosity and quest for meaning	−3.58^***^	[−5.20, −1.95]
Trivialization and wishful thinking	0.07	[−1.10, 1.23]
**Control variables**
Child-related variables		
Age (years)	0.58^***^	[0.34, 0.81]
Gender (f/m)	0.50	[−1.38, 2.38]
Parent-related variables		
Gender (f/m)	−2.31	[−4.76, 0.15]
Psychological symptom burden (BSI)	4.56^***^	[2.74, 6.37]
**Random effects**
Variance of residuals	46.57^***^	[35.40, 61.26]
Variance of intercepts	19.14^**^	[9.43, 38.83]
ICC	0.29	
**Model fit**
Deviance	2007.69	
χ^2^(df)	91.77 (9)^***^	
BIC	2018.96	
*R*^2^child level	0.14	
*R*^2^family level	0.40	

*n = 290 children clustered in 195 families. ICC, intraclass correlation coefficient, BIC, Bayesian information criterion, CI, confidence interval; all continuous regressors were grand-mean centered; analyses were conducted with a linear mixed model and were based on raw data; FQCI, Freiburg Questionnaire of Coping with Illness, BSI, Brief Symptom Inventory;*

* *p < 0.05;*

** *p < 0.01;*

*** *p < 0.001*.

**Table 4:** Association of parental coping with mental illness and externalizing problems in children and adolescents.

**Effects**	**Coefficients**	**95% CI**
**Fixed effects**
Intercept	12.06^***^	[10.45, 13.67]
Parental coping (FQCI)		
Depressed processing style	1.46	[−0.30, 3.23]
Active problem-oriented coping	0.36	[−1.36, 2.08]
Distraction and self-growth	1.39	[−0.59, 3.37]
Religiosity and quest for meaning	−1.90^*^	[−3.63, −0.17]
Trivialization and wishful thinking	0.91	[−0.32, 2.15]
**Control variables**
Child-related variables		
Age (years)	−0.35^*^	[−0.61, −0.08]
Gender (f/m)	2.82^*^	[0.68, 4.95]
Parent-related variables		
Gender (f/m)	−3.28^*^	[−5.89, −0.67]
Psychological symptom burden (BSI)	2.65^**^	[0.72, 4.58]
**Random effects**		
Variance of residuals	72.31^***^	[56.02, 93.33]
Variance of intercepts	9.28	[1.70, 50.60]
ICC	0.11	
**Model fit**
Deviance	2076.88	
χ^2^(df)	62.72 (9)^***^	
BIC	2088.15	
*R*^2^child level	0.05	
*R*^2^family level	0.53	

*n = 290 children clustered in 195 families. ICC, intraclass correlation coefficient, BIC, Bayesian information criterion, CI, confidence interval; all continuous regressors were grand-mean centered; analyses were conducted with a linear mixed model and were based on raw data; FQCI, Freiburg Questionnaire of Coping with Illness, BSI, Brief Symptom Inventory;*

* *p < 0.05;*

** *p < 0.01;*

*** *p < 0.001*.

**Table 5:** Association of parental coping with mental illness and children's diagnosis of a mental health disorder.

**Effects**	**OR**	**95% CI**
**Fixed effects**		
Intercept	0.71	[0.48, 1.05]
Parental coping (FQCI)
Depressed processing style	0.74	[0.48, 1.12]
Active problem-oriented coping	1.08	[0.72, 1.62]
Distraction and self-growth	1.22	[0.78, 1.93]
Religiosity and quest for meaning	0.58^*^	[0.38, 0.88]
Trivialization and wishful thinking	1.15	[0.86, 1.54]
**Control variables**
Child-related variables		
Age (years)	0.98	[0.92, 1.04]
Gender (f/m)	1.30	[0.78, 2.18]
Parent-related variables		
Gender (f/m)	0.83	[0.46, 1.51]
Psychological symptom burden (BSI)	1.84^**^	[1.17, 2.91]
**Random effects**		
Variance of intercepts	0.01	[0.00, 0.96]
**Model fit**
Deviance	350.44	
χ^2^(df)	17.81 (9)^*^	
BIC	412.0	

*n = 269 children clustered in 182 families. BIC, Bayesian information criterion, OR, odds ratio, CI, confidence interval; all continuous regressors were grand-mean centered; analyses were conducted with a binary logistic linear mixed model and were based on raw data; FQCI, Freiburg Questionnaire of Coping with Illness, BSI, Brief Symptom Inventory;*

* *p < 0.05;*

** *p < 0.01*.

Externalizing behavior was significantly related to religiosity and quest for meaning with higher levels of this coping style being related to fewer externalizing symptoms. Among the control variables, child age and child and parent gender, as well as parental symptom burden were associated with externalizing symptoms from the parents' perspective. Older children displayed significantly fewer externalizing symptoms. Boys showed significantly more externalizing behavior than girls. Male gender of the mentally ill parent was associated with less externalizing symptoms in children. A higher psychological symptom burden in parents was significantly associated with more externalizing behavior in children.

There was a significant negative association between children's diagnosis of a mental health disorder and parental coping in terms of religiosity and quest for meaning. Increased religiosity and quest for meaning was related to lower odds of a mental health disorder in children. Further, the control variable psychological symptom burden in parents was significantly related to children's diagnosis with higher levels of parental psychological symptom burden being related to higher odds of children's mental health diagnosis.

## Discussion

Across all analyses, religiosity and quest for meaning was significantly associated with fewer behavioral and emotional symptoms and mental health diagnoses in children. On the contrary, a depressed processing style was related to an increase of child internalizing symptoms. For the other investigated parental coping styles—active problem-oriented coping, distraction, and self-growth, and trivialization and wishful thinking—there was no significant association with mental health in children. In our analyses, we controlled for several child- and parent-related variables. Younger children displayed more externalizing and older children more internalizing symptoms. Boys showed more externalizing symptoms than girls. Mentally ill fathers reported less externalizing symptoms in their children than mentally ill mothers did. Higher parental psychological symptom burden was consistently associated with more mental health symptoms and a higher odds of a mental health disorder in children.

Our finding that a depressed coping style in mentally ill parents is related to more mental health symptoms in their children from the parents' perspective is in line with previous research (54). Across a variety of studies, depressive coping strategies have been associated with poor psychological functioning (68, 69). Adults with a mental illness are especially prone to such ineffective coping strategies (70). According to Goodman and Gotlib (71) exposure to maladaptive cognitions and behaviors is one mechanism by which the risk for depression is transmitted from parents to their offspring and children acquire those cognitions and behaviors through social learning. Being exposed to depressive ways of dealing with one's mental illness may likewise lead to the adoption of such strategies in children (18). Maladaptive coping in terms of rumination and intrusive thoughts has been linked to mental health symptoms in COPMI in previous research (72).

However, our finding that religiosity and quest for meaning in the parents is associated with fewer symptoms in their children is in contrast to the findings of Wiegand-Grefe et al. (54) and Wiegand-Grefe et al. (53). In both studies, parents reported more mental health symptoms in children when parents with mental illness scored higher on religiosity and quest for meaning. Research results on the association between religious coping and psychological outcomes are inconsistent. Tepper et al. (73) found that the amount of time that patients with severe mental illnesses devoted to religious coping was negatively related to their symptom severity and concluded that religious coping may serve as a potentially effective method of dealing with mental illness. In their meta-analysis, Ano and Vasconcelles (74) distinguish between positive forms (e.g., benevolent religious reappraisals) and negative forms (e.g., passive religious deferral) of religious coping with positive forms being related to good psychological adjustment and negative forms to poor outcomes like increased depression and anxiety. In our analyses, we did not specifically differentiate between positive and negative forms of religious coping. Items of the FQCI scale like “Seeking comfort in religious faith” and “Wanting to do good for others” are supposedly more related to the positive concept of religious coping, which might be associated with better adjustment in parents and in turn have a beneficial effect on their assessments of children's mental health. Given the relatively low internal consistency of the respective scale in this study, future research would further benefit from psychometric evaluations of the FQCI in clinal samples of parents with mental illnesses.

We could not replicate the findings of Wiegand-Grefe et al. (53) that active-problem oriented coping is associated with fewer psychological difficulties in children. One reason might be, that whereas Wiegand-Grefe et al. (53) analyzed the bivariate associations between coping and child mental health, we considered the role of several coping strategies simultaneously. An active-problem oriented coping style in mentally ill parents may have a smaller impact on child mental health compared to depressive coping and religiosity and quest for meaning. Nevertheless, studies indicate that active-problem oriented coping is a relevant adaptive coping strategy for one's own mental health (34, 38) and its role for child mental health should be further investigated.

Regarding control variables, our results are consistent with those of previous research regarding the impact of parental symptom burden (19, 22) as well as child age and gender (75). Likewise, maternal mental illness has been associated with slightly more severe behavior problems in children than paternal mental illness (76). Our study also adds to previous research in showing that the offspring of mentally ill parents are at increased risk of psychological symptoms (4, 5). Our sample of COPMI significantly differed from the norm population of the CBCL and showed elevated levels of mental health symptoms—particularly internalizing symptoms. Further, around two-fifth of the children met ICD-10 criteria for a mental disorder.

### Limitations

This study had several limitations. First, analyses were conducted in a cross-sectional study design which does not allow to draw conclusions on the temporal relationship between exposure and outcome variables. Parental coping predicted child psychopathology in our analyses, but there might also be a reverse effect. Further, a growing body of longitudinal research suggests there is a bidirectional relationship between parental and child psychopathology (77). Thus, longitudinal data are needed to validate our results. Second, we included self- and proxy-reports of parents with mental illness to assess the parents' coping behavior and the children's mental health. We further incorporated a more objective outcome measure of children's diagnoses from diagnostic interviews conducted by clinicians. However, including further rating perspectives like children's self-ratings or proxy-reports by partners may lead to a more complete picture. This is especially relevant since previous studies indicate that the reports of parents with mental illness might be biased due to the psychopathology (78). Third, regarding characteristics of the parental mental illness, we only controlled for psychological symptom burden and did not differentiate between illness duration, chronicity, and mental diagnoses. Or findings might be more representative of families affected by parental affective disorders as this was the most prevalent disorder in our sample. Also, parental mental diagnoses were not assessed with standardized diagnostic interviews, which should be applied in future studies to enhance diagnostic accuracy. Future studies may also conduct standardized diagnostic interviews with the other parent and assess their coping behavior as this may also have an influence on child mental health.

### Conclusion and Implications

Our results indicate that parental coping with a mental illness is related to the mental health of their children. Because research on this particular topic is scarce, our analyses should be replicated in future studies. This is especially true for the effect of parental religious coping since our results contradict those of previous findings (53, 54). Researchers should further increase the understanding of relevant risk and protective factors for COPMI. As a substantial amount of variance in child emotional and behavioral symptoms remained unexplained in our analyses, additional variables should be included in future studies. For example, children's own coping behavior might be considered, which seems especially relevant for the adjustment to parental illness and the children's mental health (47, 72, 79, 80). An interesting line of research would also be to investigate the relationship between parental coping strategies and children's coping strategies in families affected by parental mental illness. Regarding the clinical implications, coping strategies are a central target in many psychotherapeutic interventions (30, 31). Our results indicate that adaptive coping is not only relevant for the mental health of patients but also has an impact on the mental health of their children. Children may benefit from the positive effects of coping interventions offered for parents with mental illness. Illness-related coping should therefore be considered in preventive strategies for COPMI. Professionals should be provided with more information regarding effective coping with parental mental illness. Finally, our results again highlight the at-risk status of COPMI for the development of psychopathology and the need to further improve the provision of support for these children, adolescents, and their families.

## Data Availability Statement

The raw data supporting the conclusions of this article will be made available by the authors, without undue reservation.

## Ethics Statement

The studies involving human participants were reviewed and approved by the Ethics Committee of the Regional Medical Association (Hamburg) approved the study under the number PV4744. Written informed consent was obtained from all adult participants. Written assents of children (<18 years) and their parents' permission were provided. Written informed consent to participate in this study was provided by the participants' legal guardian/next of kin.

## Author Contributions

MSe and AR were responsible for the conceptualization, formal analysis of the present research objectives, as well as for visualizing, and writing and editing the original draft. The latter was critically reviewed by AP-C, SW-G, MSt, and AD data were provided by the CHIMPS study group with SW-G being the principal researcher. SW-G, RK, and ML were significantly involved in the conception of the CHIMPS project with methodological support provided by AD and KW and study advisory by AP-C, SW-G, and BA coordinated the multi-center project and were responsible together with ML, SW, RK, and MSt for recruitment. SW-G coordinated the evaluation of the overall CHIMPS project, supported by MB. All authors have read and agreed to the published version of the manuscript.

## Funding

This research was funded by the German Federal Ministry of Education and Research (BMBF), Grant No. 01GY1337.

## Conflict of Interest

The authors declare that the research was conducted in the absence of any commercial or financial relationships that could be construed as a potential conflict of interest.

## Publisher's Note

All claims expressed in this article are solely those of the authors and do not necessarily represent those of their affiliated organizations, or those of the publisher, the editors and the reviewers. Any product that may be evaluated in this article, or claim that may be made by its manufacturer, is not guaranteed or endorsed by the publisher.
